# Enhancing Macrophage Drug Delivery Efficiency via Co-Localization of Cells and Drug-Loaded Microcarriers in 3D Resonant Ultrasound Field

**DOI:** 10.1371/journal.pone.0135321

**Published:** 2015-08-12

**Authors:** Yu-Hsiang Lee, Zhen-Yu Wu

**Affiliations:** Graduate Institute of Biomedical Engineering, National Central University, Taoyuan City, Taiwan, R.O.C; Brandeis University, UNITED STATES

## Abstract

In this study, a novel synthetic 3D molecular transfer system which involved the use of model drug calcein-AM-encapsulated poly(lactic-co-glycolic acid) microspheres (CAPMs) and resonant ultrasound field (RUF) with frequency of 1 MHz and output intensity of 0.5 W/cm^2^ for macrophage drug delivery was explored. We hypothesized that the efficiency of CAPMs-mediated drug delivery aided by RUF can be promoted by increasing the contact opportunities between cells and the micrometer-sized drug carriers due to effects of acoustic radiation forces generated by RUF. Through the fluoromicroscopic and flow cytometric analyses, our results showed that both DH82 macrophages and CAPMs can be quickly brought to acoustic pressure nodes within 20 sec under RUF exposure, and were consequently aggregated throughout the time course. The efficacy of cellular uptake of CAPMs was enhanced with increased RUF exposure time where a 3-fold augmentation (*P* < 0.05) was obtained after 15 min of RUF exposure. We further demonstrated that the enhanced CAPM delivery efficiency was mainly contributed by the co-localization of cells and CAPMs resulting from the application of the RUF, rather than from sonoporation. In summary, the developed molecular delivery approach provides a feasible means for macrophage drug delivery.

## Introduction

Macrophages are intrinsically involved in the inflammatory process through the production of proinflammatory and/or proangiogenic mediators [1], as well as conduction of phagocytosis via opsonin receptor-dependent/independent mechanisms [2]. Since inflammation is a key driver of the onset and/or progression of various diseases such as cancer [3, 4], tuberculosis [5], rheumatoid arthritis [6], and HIV infection [7, 8], strategies aimed at targeting the macrophages have gained increasing attention in the field of drug/gene delivery over the past decade. However, macrophages have long been reputed to be difficult targets because of limited efficiency of molecular transfer and rapid degradation of internalized chemicals and/or biologicals caused by the innate endolysosomal system [9] that have seriously hampered the macrophage-related applications in clinic. Therefore, development of an efficient and biomolecule-protecting means of macrophage transfection is one of the most desirable goals for immunotherapy. Thus far, a number of approaches for delivering exogenous bioactive molecules (e.g., nucleic acids and/or proteins) into macrophages have been widely reported including use of synthetic drug vehicles such as micro-/nanoparticles [10, 11], liposomes [12], carbon nanotubes [13], and dendrimers [14], and utilization of physical means such as electroporation [15] or sonoporation [16]. Although some success has been achieved through the use of these methods, several drawbacks, such as insufficient transfection rates, serious cell damage, and/or lack of scalable capacity still remain obstacles for their practical use. To circumvent these issues, a synthetic molecular transfer system involving use of drug-loaded poly(lactic-co-glycolic acid) (PLGA) microspheres and resonant ultrasound field (RUF) was explored in this study.

Since micrometer-sized PLGA particles can be efficiently phagocytosed by macrophages, PLGA microspheres have been widely used as drug carriers for immunological applications both *in vitro* and *in vivo* [17, 18]. Furthermore, the degradation of PLGA can be modulated by adjusting the molecular weight and/or ratio of lactide to glycolide in the PLGA molecules that renders a feature of controlled drug release to the polymeric particles whereas the exact release efficiency is additionally dependent upon the type of drug encapsulated [19]. In this study, substrate calcein acetoxymethylester (calcein-AM) was employed as the model drug which is a non-fluorescent and cell-permeable compound. After entering the cells, calcein-AM can be hydrolyzed by endogenous esterase and converted into calcein, a polyanionic derivative of fluorescein which can be retained in cells and easily detected by fluorescent microscopy. This property has led to widespread use of calcein-AM as a versatile dye in various cell-based assays, including drug delivery study [20].

The motions of micrometer-sized particles (e.g., mammalian cells) in non-cavitation, MHz-frequency-ranged RUF have been known to be driven by both the primary (*F*_1_) and the secondary (*F*_2_) acoustic radiation forces [21, 22] that

$$F_{1}=-\frac{\pi P_{0}{}^{2}V\beta_{0}}{2\lambda }\times \left(\frac{5\rho_{p}-2\rho_{0}}{2\rho_{p}+\rho_{0}}-\frac{\beta_{p}}{\beta_{0}}\right)\times \text{sin}\left(\frac{4\pi z}{\lambda }\right)$$ (1)

and

**Fig 1:**
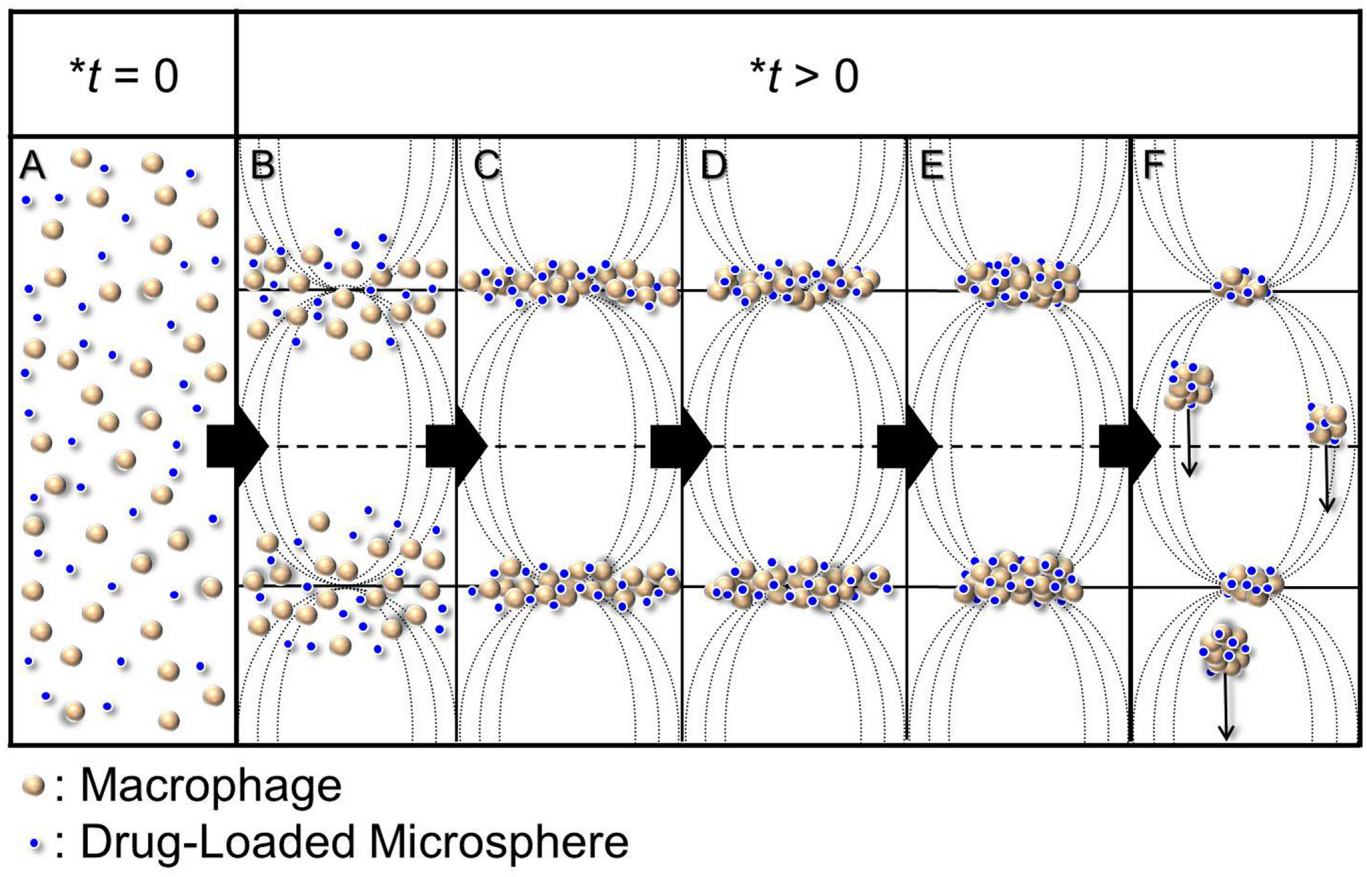
Schematic diagram of RUF-mediated co-localization of macrophages and drug-loaded microspheres in 3D. Driven by the primary acoustic radiation force, all of the microparticles, including the cells and drug-loaded microspheres, can move to the pressure nodes and then aggregate such that the columns of clumps striate at half-wavelength intervals in the direction of the ultrasonic waves (A—C). Due to the effects of the secondary acoustic radiation force, these microparticle-composed clusters are then compressed and the slender bands are turned into short and thick as represented from C to E, whereby the cells and drug carriers are placed in close contact with one another. When the weight of enlarged agglomerates is higher than the acoustic radiation force imposed, the clumps fall down and the suspended configurations collapse (F). *t represents the time of RUF exposure.

F2=4πR60[(ρp−ρ0)2(3cos2θ−1)6ρ0d4v2−ω2ρ0(βP−β0)29d2P2] (2)

where *P*_*0*_ is the peak pressure amplitude of the ultrasonic standing wave; *λ* is sound wavelength; *V* is the volume of the particles; *z* represents the propagating distance of the ultrasonic wave which is perpendicular to the pressure nodal planes; *ρ*_*p*_ and *β*_*p*_ are the density and compressibility, respectively, of the particles and *ρ*_*0*_ and *β*_*0*_ denote the density and compressibility, respectively, of the surrounding bulk phase; *R*_0_ is the radius of the particle; *d* is the distance between particles; *θ* is the angle between the centerline of the particle and the propagating direction of the sound wave; *ω* is the angular velocity; *v* denotes the particle velocity; and *p* is the amplitude of acoustic pressure at the pressure nodes. Theoretically, microparticles can arrive at acoustic pressure nodes within seconds [23], which suggests that all the cells and drug microcarriers can meet in 3D quickly under RUF exposure. Therefore, we hypothesized that we could utilize the drug-loaded microcarriers and RUF to enhance the efficiency of drug delivery to macrophages by increasing the contact opportunities between cells and drug vehicles as illustrated in Fig 1. We reasoned that under RUF exposure, both cells and calcein-AM-loaded PLGA microspheres (CAPMs) can be co-localized at the pressure nodes and kept in close contact by the acoustic radiation forces that allow the CAPMs to be phagocytosed efficiently. In this study, the efficacy of RUF on CAPM delivery rate and the mechanism of the effect were comprehensively investigated.

## Materials and Methods

### Acoustic apparatus setup

**Fig 2:**
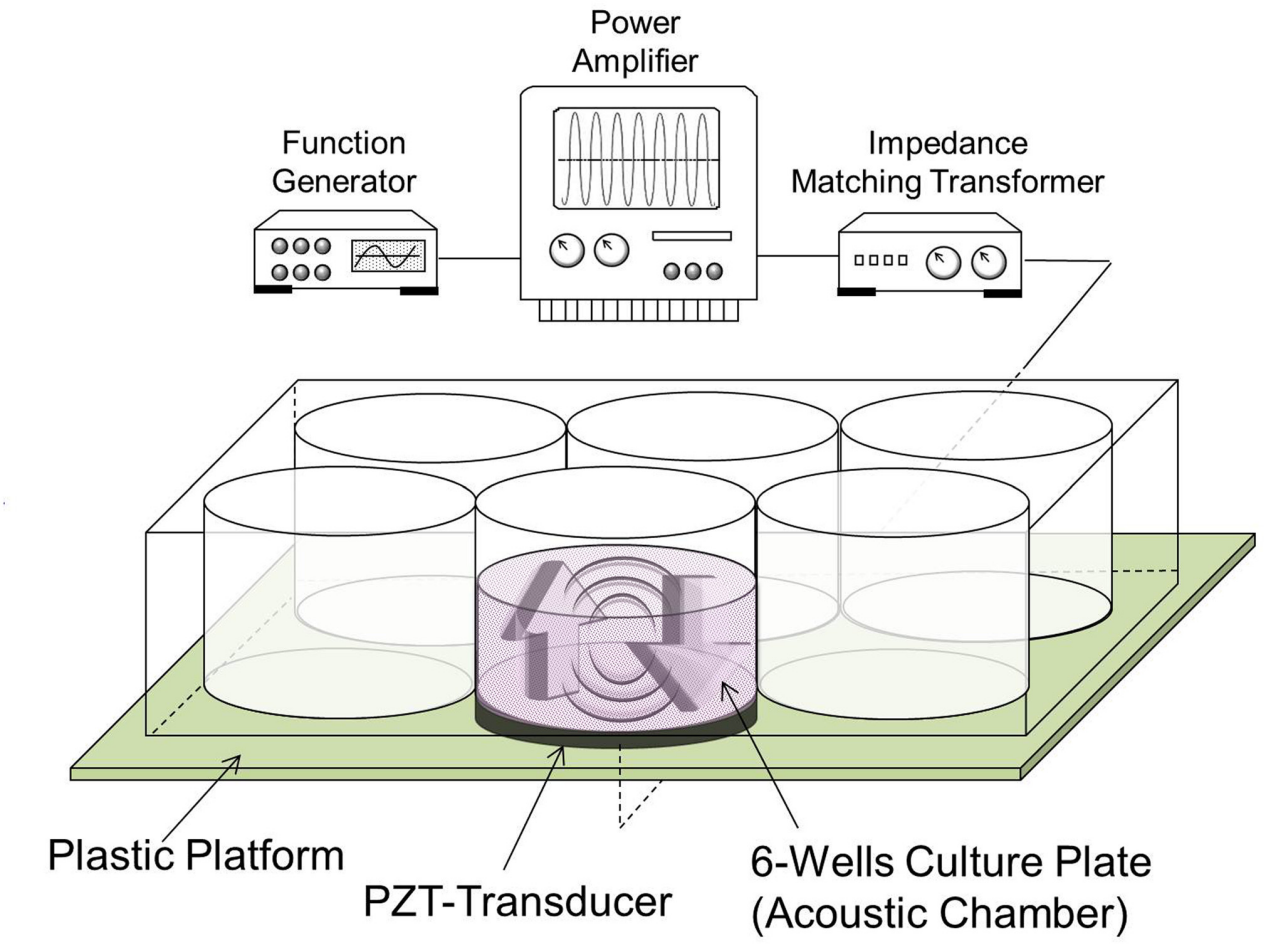
Schematic diagram of the acoustic setup. The PZT transducer mounted on a plastic platform was connected to the ultrasound system including a function generator, a power amplifier, and an impedance matching transformer. The sonolucent six-well culture plate was used as the acoustic chamber in this study. After the cells and/or CAPMs were homogeneously distributed in the well, the RUF irradiation with frequency of 1 MHz and output intensity of 0.5 W/cm^2^ was applied for defined minutes. The two arrows represent the directions of wave propagation in which the acoustic waves were transmitted from the bottom of the well (↑) as well as reflected from the gas-liquid interface at the top (↓), resulting in RUF inside the chamber.

The full experimental setup is illustrated in Fig 2. Continuous 1-MHz sinusoidal waves with output intensity of 0.5 W/cm^2^ were generated from a function generator (FG708S DDS; MOTECH INDUSTRIES, Taiwan ROC) and transferred through an amplifier (Model 7500; KROHN-HITE, Brockton, MA) and an impedance matching transformer (Model MT-56; KROHN-HITE) prior to reaching the transducer. The piezoelectric transducer was made of a lead zirconate titanate (PZT) disc with 50-mm diameter and 2-mm thickness (Ceramic Transducer Design Co., LTD., Taiwan ROC) which was mounted on a plastic platform as shown in Fig 2. In this study, a sonolucent six-well culture plate with 1.0-mm thickness bottom was employed as the acoustic chamber. The RUF in the acoustic chamber was established through interference of acoustic waves transmitted from the bottom piezoelectric transducer and reflected from the upper gas-liquid interface.

### Cell culture

Canis macrophages (DH82/CRL-10389; ATCC, Rockville, MD) were cultivated in the culture flask with Minimum Essential Medium supplemented with 15% fetal bovine serum, 0.1-mM non-essential amino acids, 2-mM L-glutamine, 1-mM sodium pyruvate, and 100-U/ mL penicillin-streptomycin (all reagents from Life Technologies, Carlsbad, CA). Cell culture was conducted in a 37°C incubator balanced with 5% CO_2_ and 100% humidity.

### Preparation and characterization of CAPMs

The CAPMs were fabricated by single oil-in-water emulsification in association with a solvent evaporation approach as reported elsewhere. Briefly, 300 μL of dichloromethane containing 30 mg of PLGA (50:50, MW = 7000–17000 kDa; Sigma-Aldrich, St. Louis, MO) and 15 μg of calcein-AM (absorption wavelength = 490 nm, Trevigen Inc., Gaithersburg, MD) was added to 15 mL of polyvinyl alcohol solution (0.2 wt%), following emulsification by sonication with 60 W for 30 sec under ice bath. The emulsion solution was then stirred overnight to allow all the organic solvent to evaporate. To remove excess molecules and simultaneously reduce the size dispersity of the CAPMs, the filtration method [24] was utilized in this study. The harvested CAPMs were then lyophilized for 24 h and stored at 4°C for further use. The filtered solution was additionally centrifuged at 25000 ×g for 60 min and the supernatant was collected.

The mean size and surface charge of the CAPMs after filtration were measured using the static light scattering (SLS) technique (LA-950, HORIBA, Kyoto, Japan) and a zeta potential analyzer (SZ-100, HORIBA), respectively. The morphologies of the CAPMs were detected by scanning electron microscopy (S-800; HITACHI, Tokyo, Japan) with an accelerating voltage of 20 kV. The concentration of CAPMs (*C*) suspended in the deionized water was evaluated by the formula:

$$C\approx \frac{6\times \left(W-W_{l}\right)}{\rho \pi d_{c}{}^{3}V}$$ (3)

where *W* is the entire weight of the CAPM solution; *W*_*l*_ and *V* represent the weight and volume, respectively, of the deionized water used; *ρ* is the density of PLGA; and *d*_c_ denotes the mean diameter of the CAPMs which can be obtained from the SLS measurement. The encapsulation efficiency (EE) of calcein-AM in the CAPMs was calculated by the formula:

$$EE=\frac{W_{T}-W_{S}}{W_{T}}\times 100\%$$ (4)

Where *W*_*T*_ is the total amount of calcein-AM used for fabrication of CAPMs and *W*_*S*_ denotes the amount of calcein-AM in the supernatant (i.e., un-encapsulated calcein-AM molecules) that can be obtained according to Beer-Lambert’s law.

### Cell viability and CAPM stability after RUF exposure

To assess the effect of RUF on cell in the presence of CAPMs, a total of 1.5 × 10^6^ DH82 macrophages in the exponential growth phase were aliquoted into three acoustic chambers, and one of the three groups was mixed with 2.5 × 10^6^ CAPMs. Two wells, including the one with CAPMs, were exposed to 1-MHz RUF with output intensity of 0.5 W/cm^2^ for 15 min. After the ultrasonic treatment, cells of each group were transferred to eight culture flasks with 5 × 10^4^ cells per each. The group with neither CAPM nor RUF treatment was employed as the control. The cell viability of each setting was examined at 0, 24, and 48 h post RUF exposure, while the cell growth was continuously monitored for 7 days using hemocytometry.

To examine whether RUF exposure affects the integrity of the developed drug carriers, the size, surface charge, and morphology of the CAPMs after 15-min RUF exposure with 1 MHz and 0.5 W/cm^2^ were examined. Furthermore, the supernatant of the RUF-treated CAPMs was collected and added to DH82 cells pre-settled in the flask. After incubation at 37°C for 1 h, the expressions of green fluorescence (GF) of the cells were detected using both fluorescent microscopy and fluorospectrometry with excitation wavelength of 490 nm and emission wavelength of 515 nm. The cells treated with and without 1 ng/mL of calcein-AM were employed as the positive and negative controls, respectively, in this test.

### Cellular uptake of CAPMs under RUF exposure

On the day of experiment, 6 × 10^6^ DH82 macrophages were mixed with 3 × 10^7^ CAPMs in 30 mL of culture medium. The mixture solution was then aliquoted into six acoustic chambers and exposed to 1-MHz RUF with output intensity of 0.5 W/cm^2^ for 0, 1, 3, 5, 10, and 15 min. After incubation at 37°C for 4 h post RUF treatment, cells in each group were washed twice by ice-cold PBS and then recovered in 5 mL of culture medium. After incubation at 37°C for an additional 24 h, the GF expressions of the cells were photographed by fluorescent microscopy and analyzed by using a flow cytometer (XL-MCL; Beckman Coulter, Brea, CA) equipped with an ion laser exciting at a wave length of 488 nm.

### Statistical analysis

All data were acquired from three independent experiments and are presented as mean ± standard deviation (SD). Statistical analyses were conducted by using MedCalc software in which comparisons for one condition between two groups were performed by Student’s *t*-test with a significance level of *P* < 0.05 used throughout the study.

## Results

### Characterization of CAPMs

**Fig 3:**
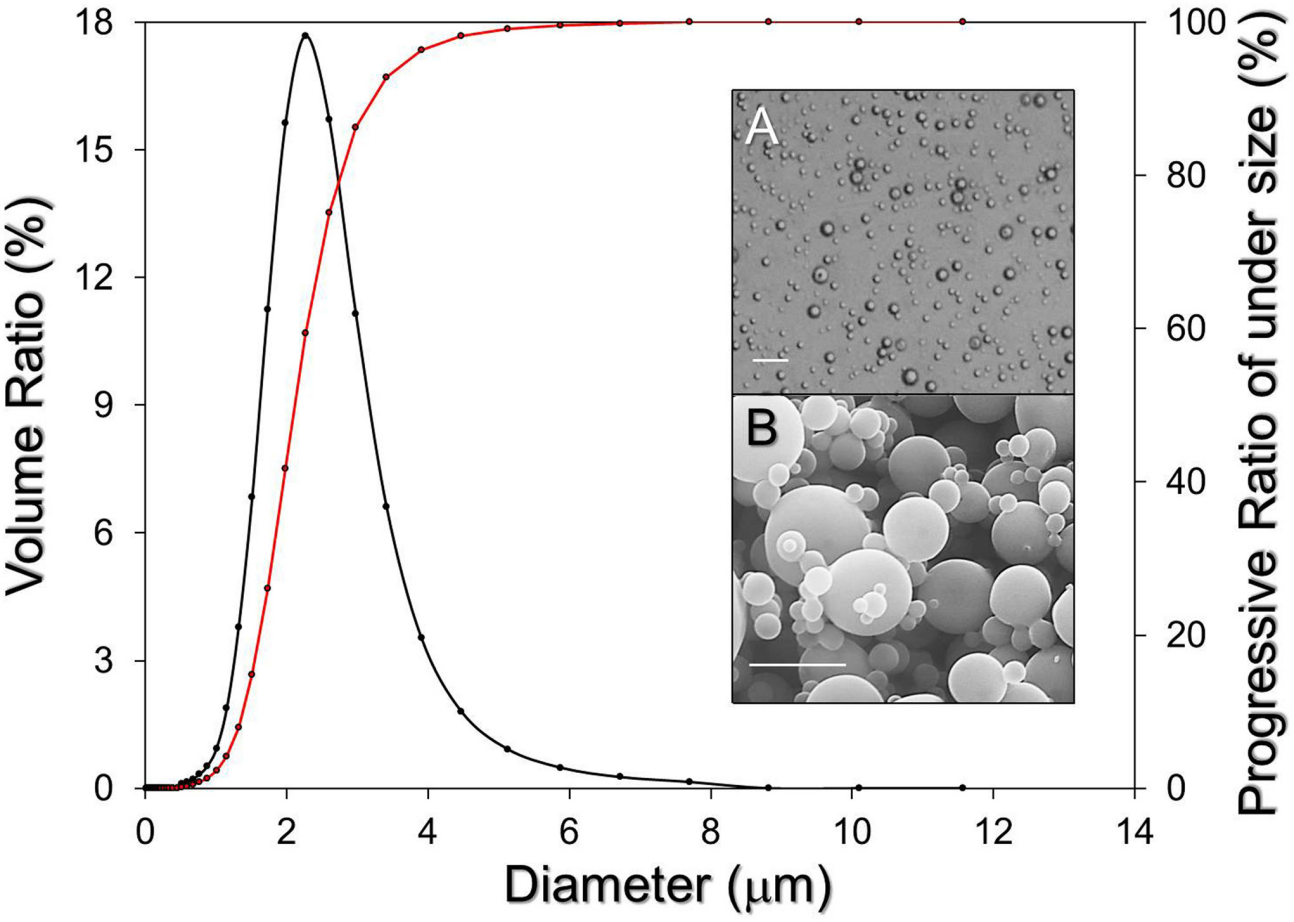
Analysis of CAPM size after the filtration process. Both size distribution (black line; left Y-axis) and progressive ratio of under size for each measured size (red line; right Y-axis) were determined by using the SLS technique. The inset images are photomicrographs of CAPMs taken by phase contrast microscopy (A; scale bar = 10 μm) and SEM (B; scale bar = 5 μm).

Fig 3 exhibits the results of the size and morphology of the CAPMs after the filtration procedures. Both the phase-contrast (Fig 3, image A) and SEM (Fig 3, image B) images show that the produced CAPMs remained intact spheroids without deformation after the fabrication process including the vacuum-aid filtration. The mean size of the CAPMs was 2.68 ± 0.07 μm in which > 90% of the particles were in the range of 1–10 μm based on the SLS analysis (Fig 3). Furthermore, the mean surface charge of the CAPMs was -91.8 ± 2.82 mV; the concentration of collected CAPMs was about 3 × 10^9^ particles/mL calculated by Eq 3, and the encapsulation efficiency of calcein-AM in the CAPMs was 76.8 ± 3.2% according to Eq 4.

**Fig 4:**
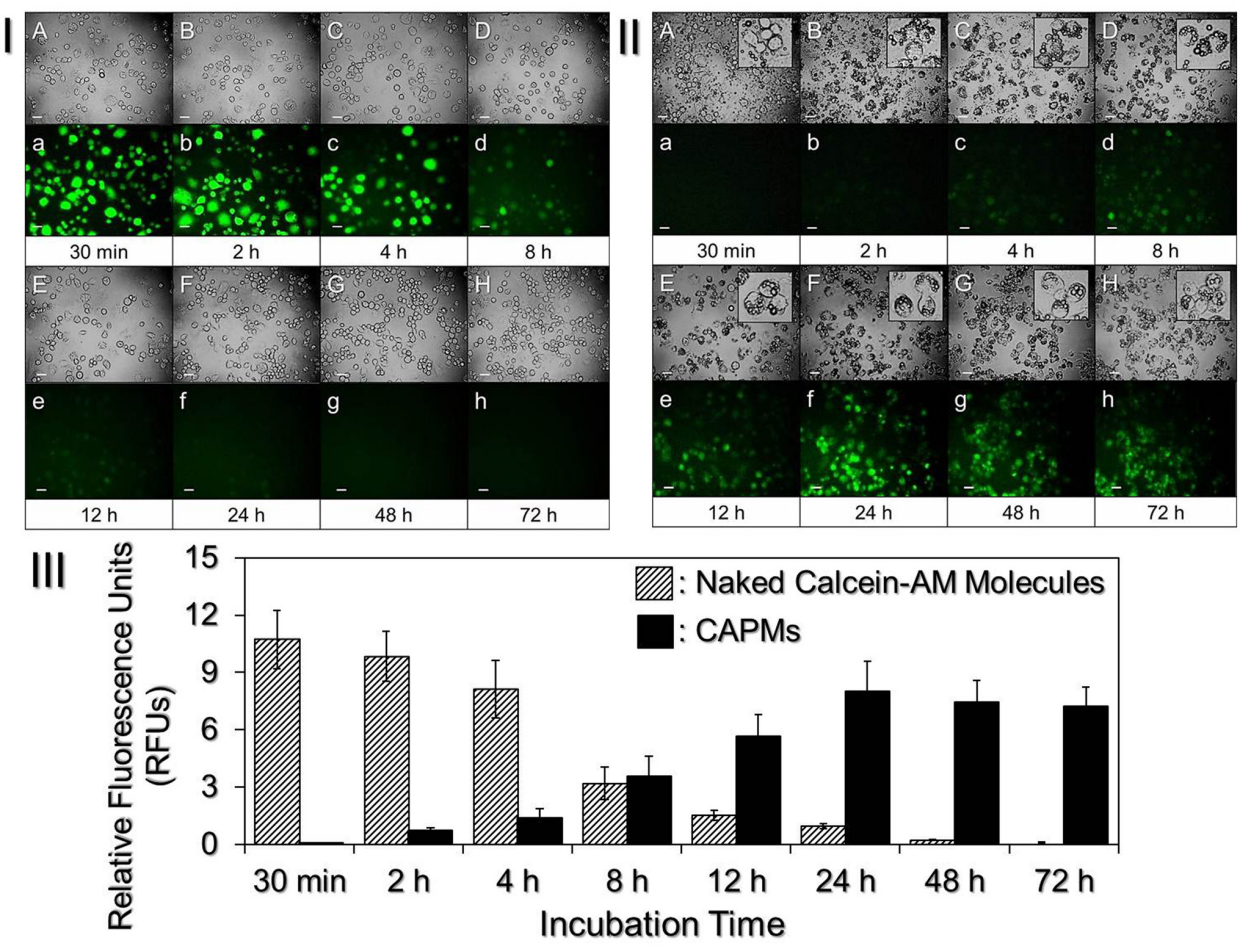
Effect of drug transfection in DH82 macrophages treated by either naked calcein-AM molecules or CAPMs. Photomicrographic images of DH82 macrophages treated by (**I**) naked calcein-AM (1 μg/mL) or (**II**) CAPMs, in which the amount ratio of cells to CAPMs was 1: 5, were taken by using phase contrast (A—H) and fluorescent microscopy (a—h) at 30 min (A/a), 2 h (B/b), 4 h (C/c), 8 h (D/d), 12 h (E/e), 24 h (F/f), 48 h (G/g), and 72 h (H/h). Scale bar = 30 μm. (**III**) Quantitative analyses of the GF expressions in the above two settings. The GF intensities of the DH82 cells at each time point were measured by fluorospectrometry performed with excitation wavelength of 490 nm and emission wavelength of 515 nm, and were presented by RFUs. Values are mean ± SD (n = 3).

The effect of drug (i.e., calcein-AM) release of the CAPMs in the phagocytic cytoplasm was assessed by comparing the GF expressions of the cells treated by CAPMs or naked calcein-AM molecules, and the results are shown in Fig 4. Based on the delayed and prolonged GF expression in the CAPMs-treated DH82 macrophages (Fig 4II and 4III), our data clearly showed that the PLGA microspheres were able to protect the encapsulated calcein-AM molecules from enzymatic digestion in the phagocytic endolysosomal system and thus the effect of GF expression was extended. This is particularly important for macrophage drug delivery because mostly the exogenous molecules are often quickly degraded by the phagocytic endolysosoms as manifested in Fig 4I.

### Progression of particles congregation at pressure nodes in RUF

**Fig 5:**
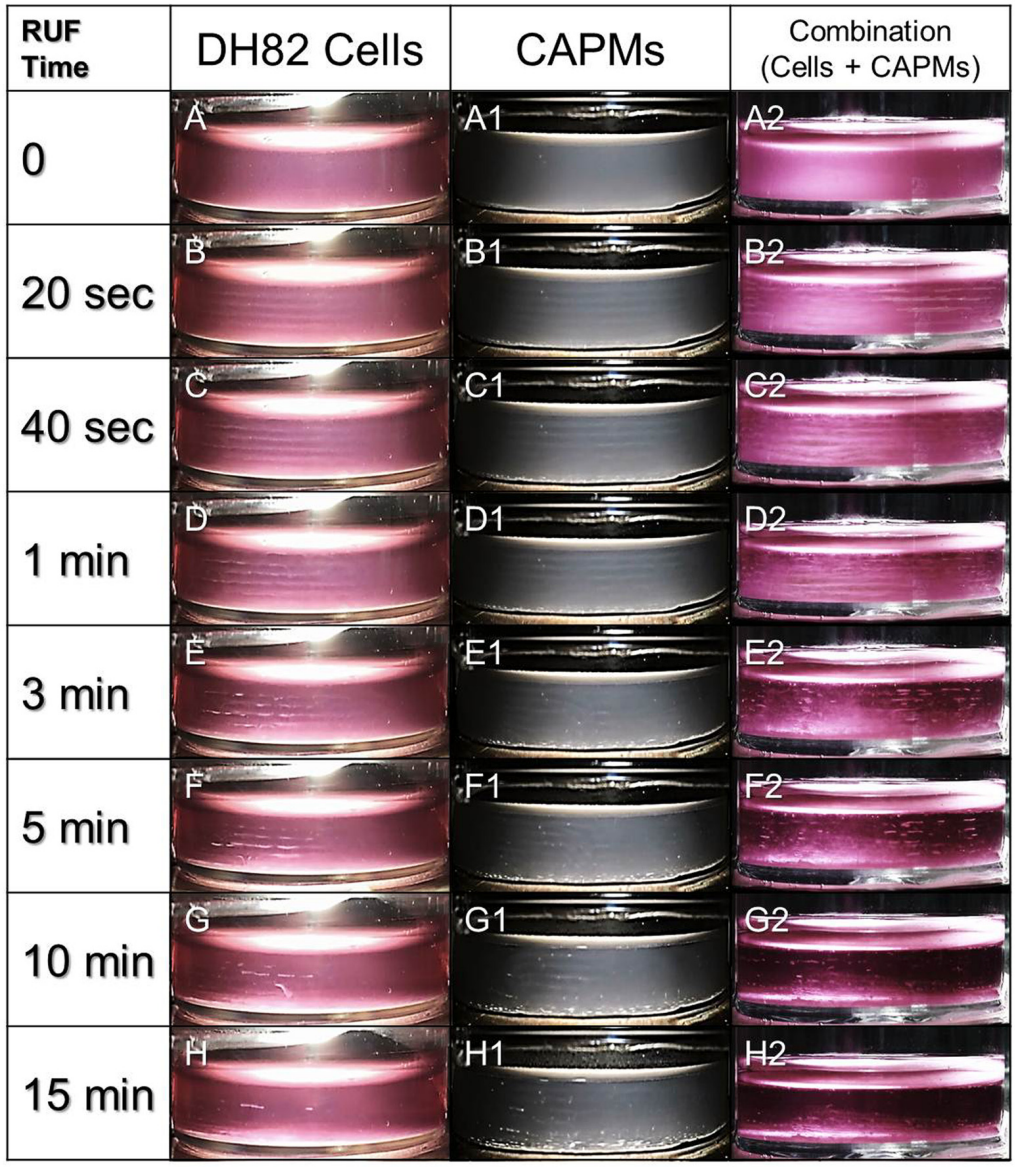
Temporal progression of the microparticle movement under RUF exposure. The motions of DH82 macrophages (A—H), CAPMs (A1—H1), or combinations of DH82 cells and CAPMs (A2—H2) under RUF exposure with frequency of 1 MHz and output intensity of 0.5 W/cm^2^ were photographed at 0, 20, 40, 60, 180, 300, 600, and 900 sec. The white stripes represent the particle agglomerates which were separated by approximately 750 μm; the half wavelength of 1-MHz sound in aqueous solution such as culture medium (for the DH82 cells and the combined particles) and PBS (for the CAPMs). The particle numbers used for settings of DH82 macrophages, CAPMs, and combinations of cells and CAPMs were 1 × 10^6^, 1 × 10^6^, and 6 × 10^6^ (cells: CAPMs = 1: 5), respectively. The volume of the medium in the acoustic chamber was 5 mL for all groups.

Fig 5 presents the formation and evolution of the aggregation of DH82 macrophages (A—H), CAPMs (A1—H1), and the combination of cells and CAPMs (A2—H2) under RUF exposure performed with 1 MHz and 0.5 W/cm^2^. It can be observed that as the RUF was initiated, the cells and microparticles moved to the pressure nodes and formed numbers of bands which were perpendicular to the direction of the ultrasonic wave. These bands were separated by ~750 μm which is about half of the acoustic wavelength in the aqueous medium. Within the first minute, the longer the RUF exposure was applied, the more distinct these striated bands became. From the 1^st^ to the 5^th^ min, the particle clusters were contracted and thickened due to the secondary acoustic radiation force [22]. These particles agglomerates started to precipitate since the 5^th^ min because the gravity of the growing agglomerates surpassed the acoustic radiation forces imposed. Based on the result of temporal progression of the cells-CAPMs combination under RUF exposure (Fig 5, A2 –H2), 15 min was determined as the maximal operation time for RUF-mediated CAPM delivery under the presented acoustic setting because most of the particle clumps fell down and very few suspended objects can be detected in the space after 15 min of RUF exposure (Fig 5, H2).

### RUF is nontoxic to cells and CAPMs

**Fig 6:**
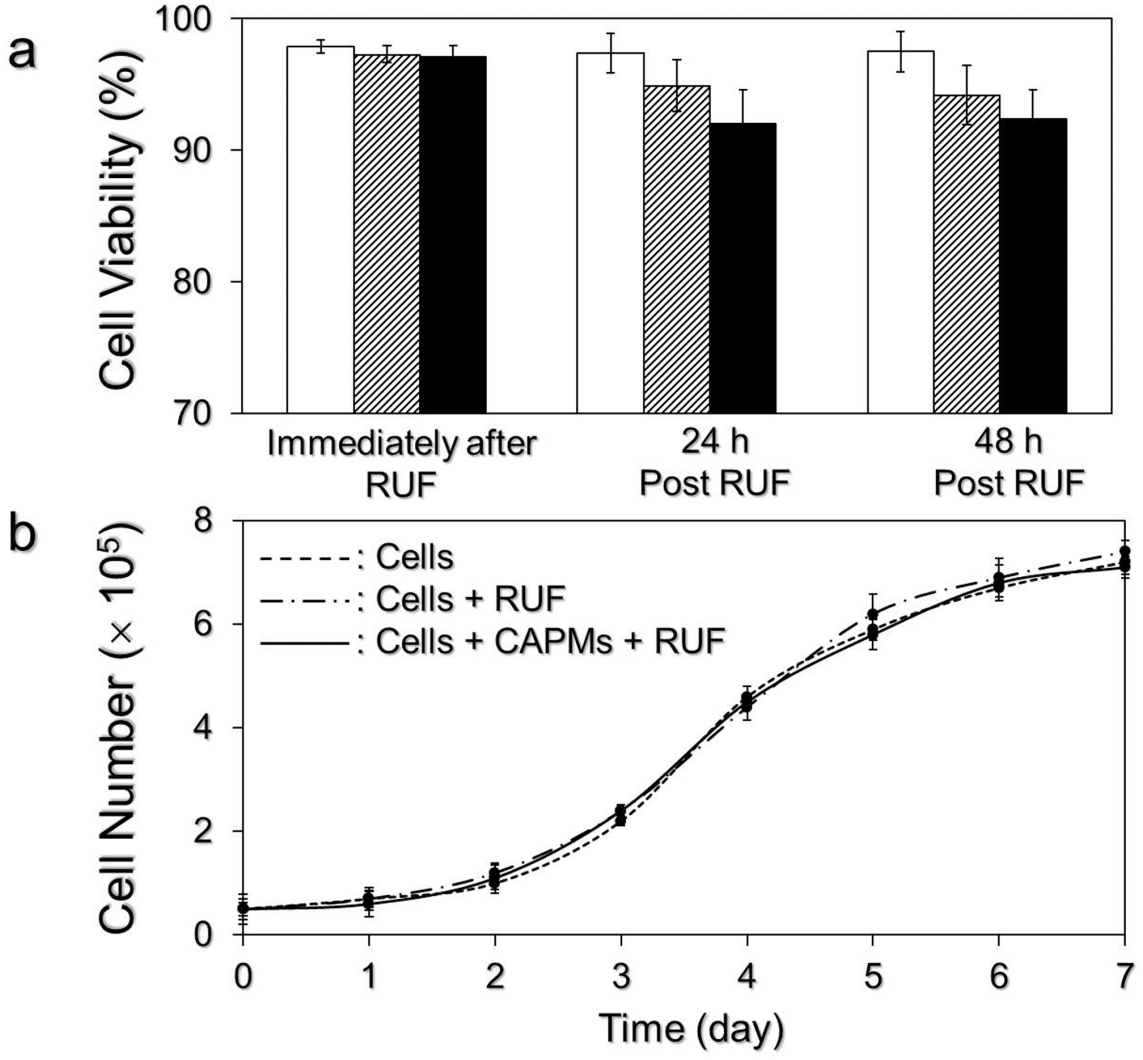
Effect of RUF on DH82 macrophages. (a) Viabilities of DH82 macrophages without RUF exposure (white bars) and RUF-treated DH82 macrophages in the presence (black bars; the amount ratio of cells to CAPMs was 1: 5) and absence (stripe bars) of CAPMs at different time points. After treated by 1-MHz RUF irradiation with output intensity of 0.5 W/cm^2^ for 15 min, viabilities of DH82 macrophages in the three settings were measured immediately and after 24- and 48-h incubation at 37°C using a hemocytometer with trypan-blue staining. Values are mean ± SD (n = 3). (b) Growth kinetic curves of DH82 macrophages of the aforementioned three groups. After RUF irradiation, all cells were cultivated at 37°C and the growth kinetic curve of each group was established through the measurements of cell numbers every 24 h for 7 days. Values are mean ± SD (n = 3).

The bioeffects of the RUF on DH82 macrophages in the presence and absence of CAPMs were evaluated through the examinations of cellular viability and proliferation after 1- MHz RUF treatment with output intensity of 0.5 W/cm^2^ for 15 min. As shown in Fig 6, both RUF-treated groups exhibited > 90% of viability within 48 h after ultrasonic treatment (Fig 6a) and similar specific growth rate of 0.456 ± 0.02 day^-1^ in 7 days which is comparable to the group with neither CAPM nor RUF as plotted in Fig 6b.

**Fig 7:**
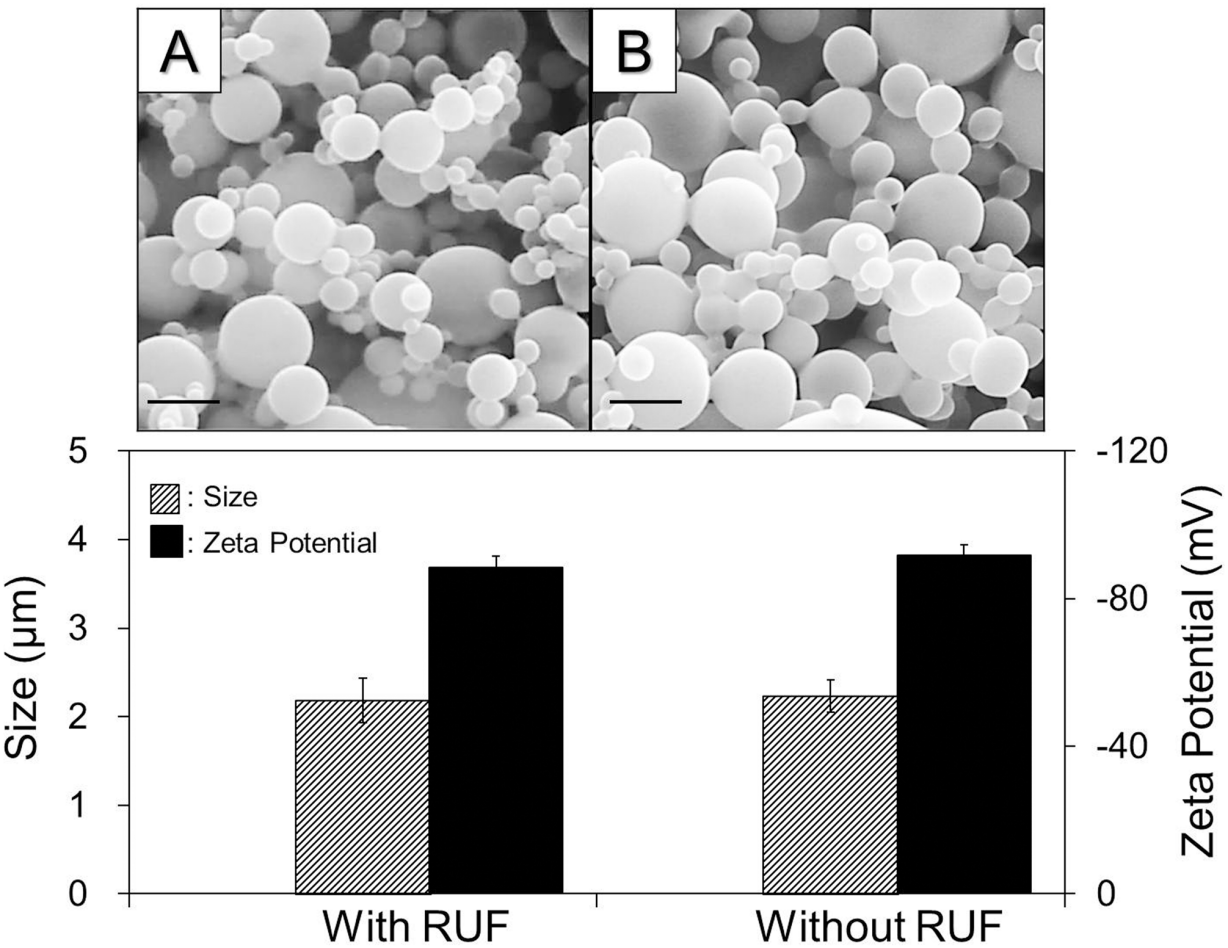
Effect of RUF irradiation on CAPMs. The upper panel exhibits the SEM images of CAPMs with (A) and without (B) RUF treatment. Scale bar = 3 μm. The bottom panel represents the quantitative analyses of the sizes (left Y-axis) and surface charges (right Y-axis) of the CAPMs with and without RUF exposure. Values are mean ± SD (n = 3). The RUF exposure was performed at frequency of 1 MHz and output intensity of 0.5 W/cm^2^ for 15 min.

**Fig 8:**
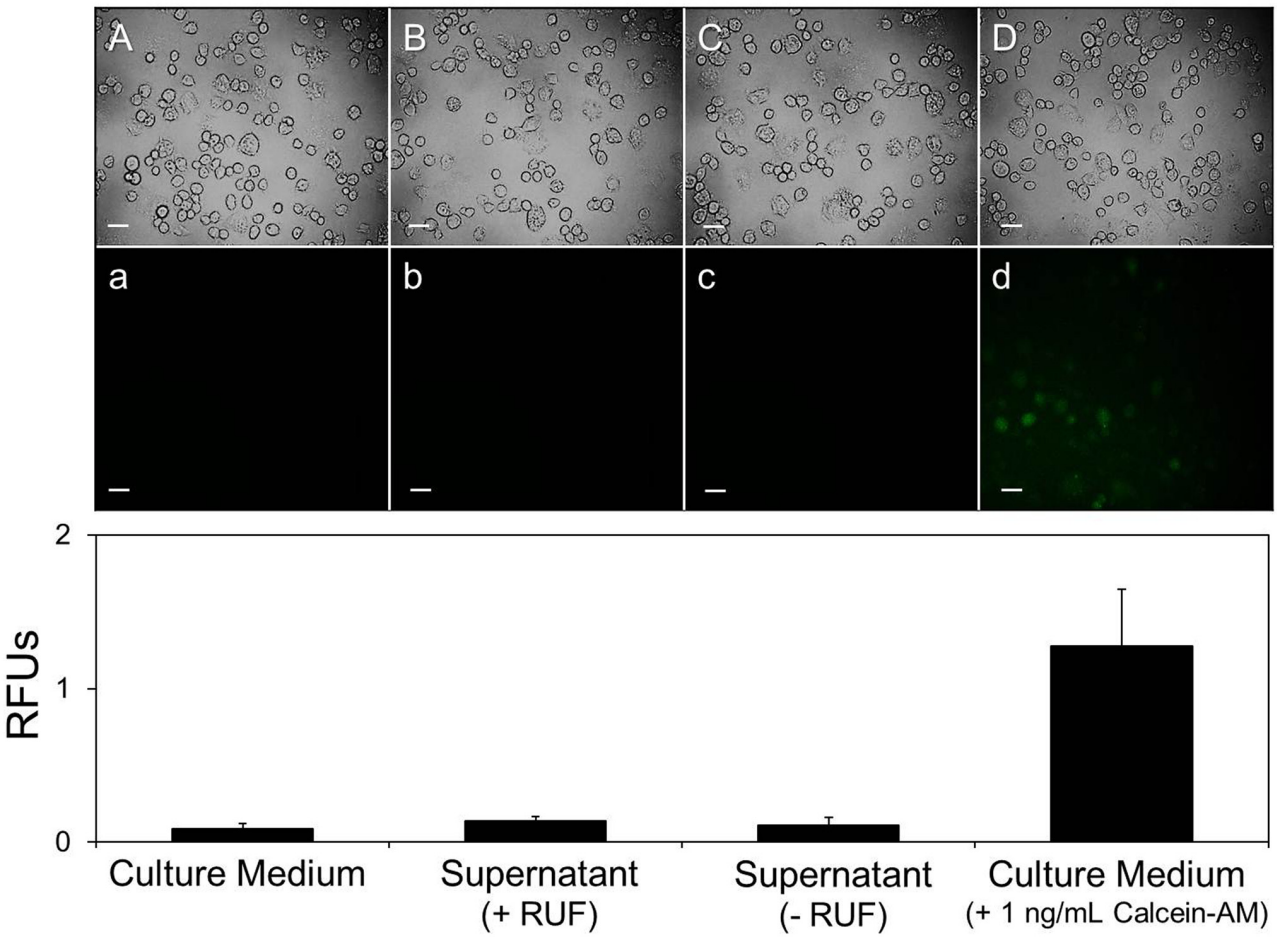
Microscopic and fluorospectrometric analyses of GF expressions of DH82 macrophages under different treatments. Micrographic images of DH82 macrophages treated by blank culture medium (A/a), supernatant of CAPMs sample with RUF (B/b), supernatant of CAPMs sample without RUF (C/c), and culture medium containing 1 ng/mL of calcein-AM (D/d) were taken using phase contrast (A—D) and fluorescent (a—d) microscopy at 1 h after treatment. Scale bar = 30 μm. Fluorescent intensities of the cells in all groups were simultaneously measured with microscopic detection by using the multi-mode microplate reader performed with excitation wave length of 490 nm and emission wave length of 515 nm and quantitatively represented by RFUs. Values are mean ± SD (n = 3).

The impact of the RUF on the CAPMs was evaluated by detecting the variations in morphology, size, and surface charge after ultrasonic exposure, as well as by measuring the level of calcein-AM released from the RUF-treated CAPMs. As shown in Fig 7, all the RUF-treated CAPMs maintained spherical shape (Fig 7, SEM image A) and exhibited similar size and zeta potential as compared to the CAPMs without RUF exposure (*P* = NS for each). In addition, the levels of GF expression for the cells cultured with supernatants isolated from the CAPMs with (Fig 8, B/b; 0.14 ± 0.03 RFUs) and without (Fig 8, C/c; 0.11 ± 0.05 RFUs) RUF were all similar to the value obtained from the blank (Fig 8, A/a; 0.09 ± 0.03 RFUs, *P* = NS for each), but significantly lower than the readout detected from the group with 1 ng/mL of calcein-AM (Fig 8, D/d; 1.28 ± 0.37 RFUs, *P* < 0.05 for each).

### RUF irradiation enhanced efficiency of cellular uptake of CAPMs

**Fig 9:**
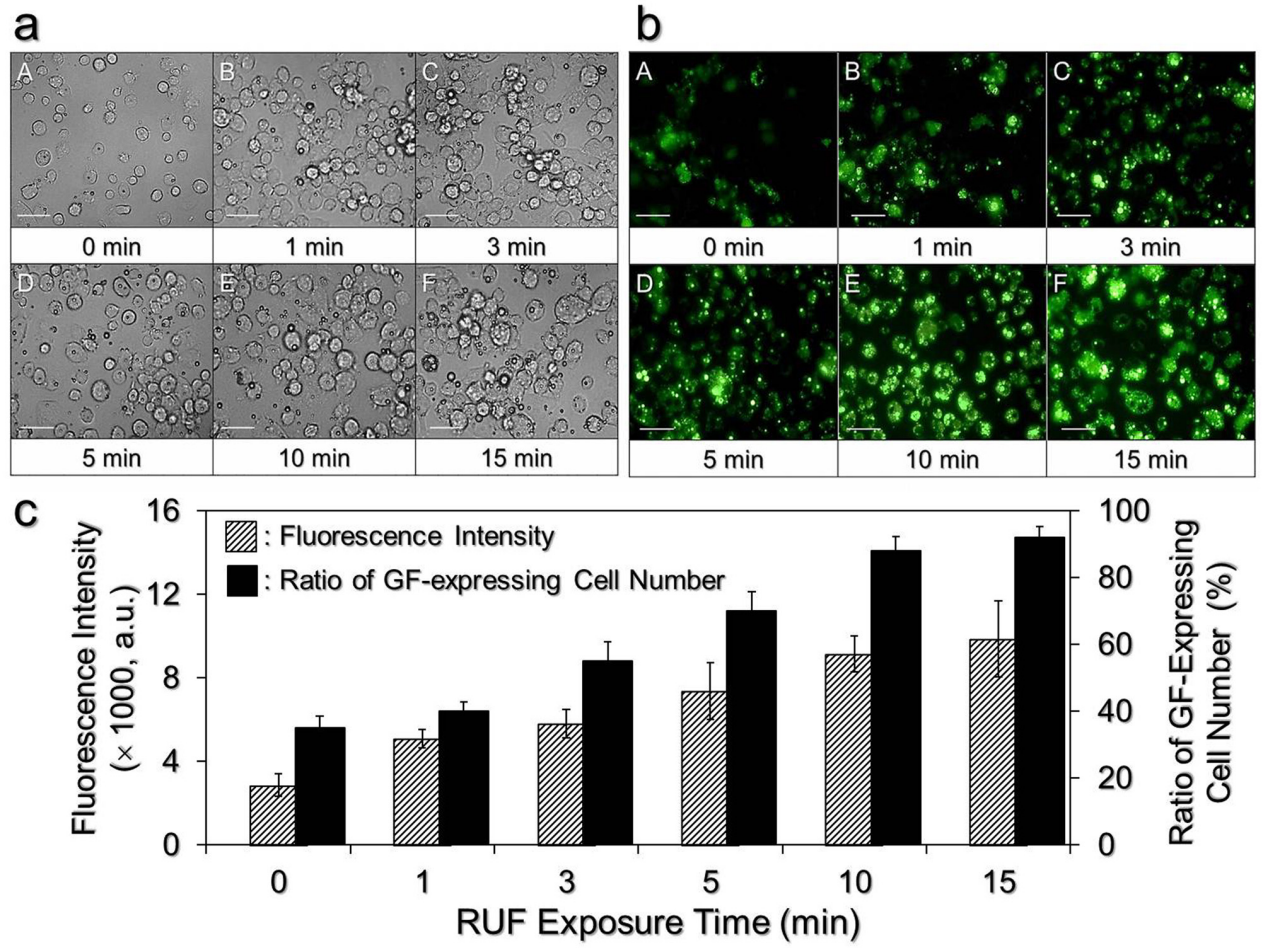
Efficiencies of CAPM delivery under different RUF exposure time. (a) Photomicrographic images of DH82 macrophages and CAPMs at 4 h after RUF exposure. The amount ratio of cells to CAPMs was 1:5 in each group and the RUF was performed with frequency of 1 MHz and output intensity of 0.5 W/cm^2^ for 0, 1, 3, 5, 10, and 15 min as indicated by A—F in the figure. After RUF exposure, the cells and CAPMs of each group were maintained at 37°C with 5% CO_2_ for 4 h, followed by washing with PBS. The photomicrographs represent the scenarios of aggregation of cells and CAPMs after washed with PBS. Scale bar = 50 μm. (b) Photomicrographic images of the aforementioned DH82 macrophages taken by fluorescent microscopy after incubation at 37°C for an additional 24 h. Scale bar = 50 μm. (c) Quantitative analyses of GF-expressing cell numbers and GF intensities of DH82 macrophages transfected by CAPMs as shown in (b). Both measurements were conducted by using the flow cytometer equipped with an ion laser exciting at the wavelength of 488 nm. The fluorescence intensity was presented by arbitrary unit (a.u.). Values are mean ± SD (n = 3).

After confirming the nontoxic effects of the RUF on the cells and CAPMs, the efficacy of RUF-mediated CAPM delivery under different exposure time was subsequently examined. Fig 9a shows the aggregating scenario of the cells and CAPMs in each group after incubation at 37°C for 4 h. It can be observed that the cells and CAPMs were indeed driven into close contact by the RUF and that the level of cells-CAPMs aggregation was enhanced with increased RUF exposure time. The drug delivery efficiency of each group was further assessed by detecting the GF expressions of the cells resulting from the hydrolysis of calcein-AM after incubation at 37°C for additional 24 h (Fig 9b). As compared to the group without RUF that only few cells expressed mild fluorescence (Fig 9b (A)), the application of RUF remarkably enhanced the efficacy of CAPM internalization and resulted in higher levels of GF expression in the cells as illustrated in Fig 9b (B)–(F). Similarly, the flow cytometric data (Fig 9c) showed that the efficiency of CAPM delivery was enhanced along with increase of RUF exposure time, by which the number of GF expressing cells and the GF intensity of the cells were increased about 2.6 and 3.4 fold, respectively, as the RUF exposure time was increased from 0 (without RUF treatment) to 15 min.

### Co-localization is the main mechanism for enhanced RUF-mediated CAPM delivery rate

**Fig 10:**
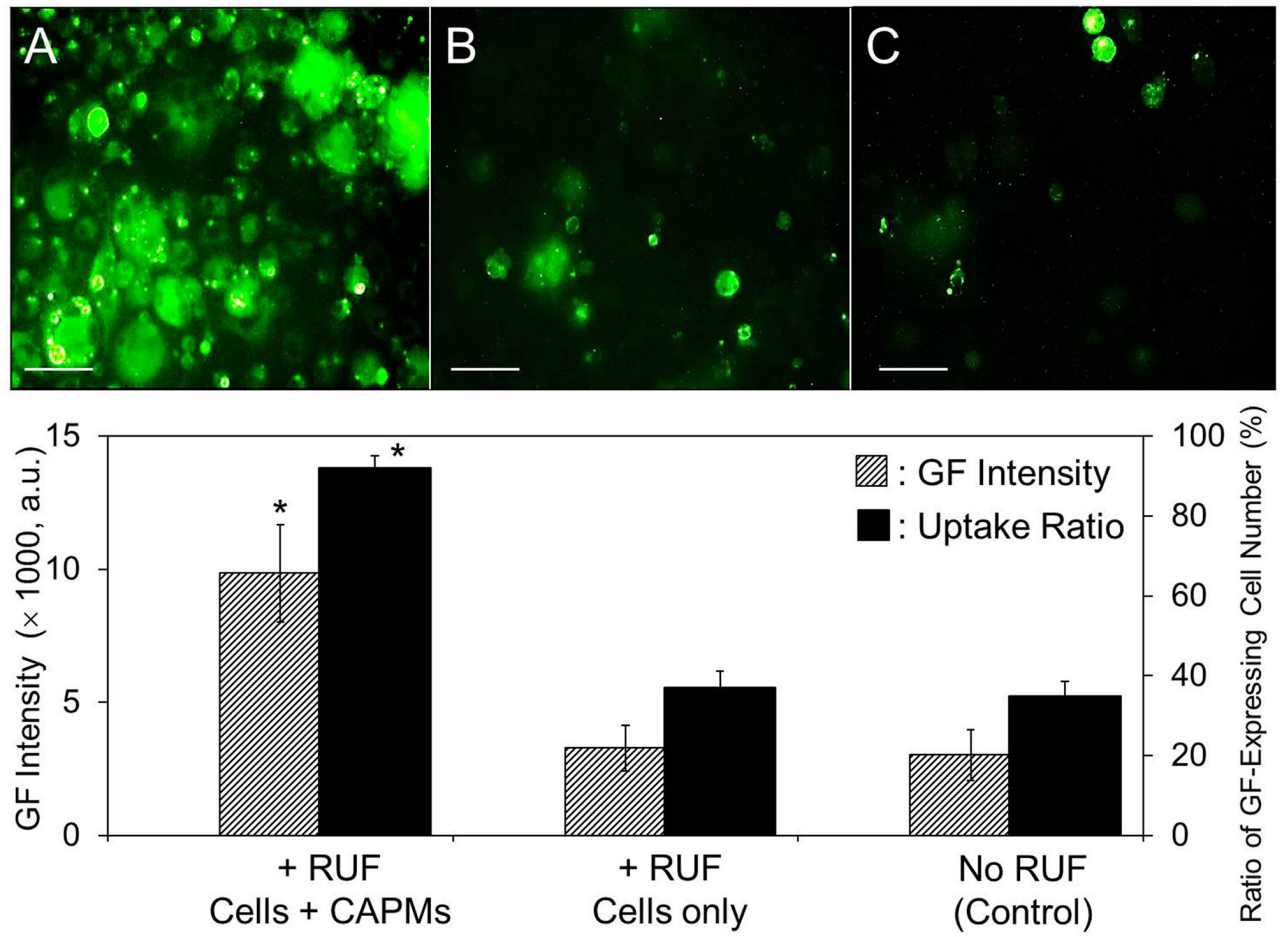
Effect of acoustic cavitation on RUF-mediated CAPM delivery. Top: Photomicrographic images of DH82 macrophages transfected under three different settings: (A) both cells and CAPMs were simultaneously treated with RUF; (B) cells were treated with RUF first and then transfected with CAPMs; and (C) CAPM delivery without RUF exposure (i.e., control group). Each set was operated in the acoustic chamber with 5-mL growth medium where the amount ratio of cells to CAPMs was 1: 5 and the RUF (for groups A and B) was performed with frequency of 1 MHz and output intensity of 0.5 W/cm^2^ for 15 min. Note that for the group B, the RUF-treated cells were decentralized by gently pipetting before addition of CAPMs. The untargeted CAPMs were removed by PBS at 4 h after RUF exposure, and the images of the GF-expressing cells were taken after incubation at 37°C for an additional 24 h using fluorescent microscopy. Scale bar = 50 μm. Bottom: The efficiency of CAPM delivery for each group based on the ratio of GF-expressing cell number (right Y-axis) and the GF intensity of cells (left X-axis) as determined by flow cytometry. Values are mean ± SD (n = 3). *P < 0.05 as compared to the control.

To verify that the enhanced CAPM delivery efficiency was mainly contributed from the co-localization of cells and CAPMs resulting from the RUF irradiation instead of from the sono-effect of the RUF (e.g., acoustic cavitation), the efficiencies of CAPM delivery of three groups that (I) both DH82 macrophages and CAPMs were simultaneously exposed to RUF, (II) DH82 macrophages were treated by RUF first and then transfected with CAPMs, and (III) CAPMs were added to DH82 macrophages without RUF (control) were examined, in which the CAPM internalization time of each group was set as 4 h and the CAPM delivery rates were examined after incubation at 37°C for an additional 24 h using fluorescent microscopy and flow cytometry. As shown in Fig 10, the ratio of GF-expressing cells and the GF intensity of the cells obtained from the group I (Fig 10, image A) significantly enhanced 2.5- and 3.0-fold (*P* < 0.05 for each), respectively, as compared to the group II (Fig 10, image B), while both experimental readouts obtained from the group II were similar to the control (Fig 10, image C; *P* = NS for each), showing that the acoustic cavitation-induced sono-effect in RUF (if there was any) was not the mechanism for the enhanced CAPM delivery efficacy.

## Discussion

Microphages have been recognized as potential vehicle for therapeutics delivery since they play an essential role in onset and/or progression of various diseases. Although quite a few strategies of macrophage drug delivery have been reported in the past decades [10–16], several drawbacks, such as insufficient transfection rates, serious cell damage, and/or lack of scalable capacity still remain obstacles for their practical use. To overcome these challenges, a synthetic molecular transfer system consisting of PLGA drug microcarriers (i.e., CAPM) and RUF operation was developed in this study.

In the developed molecular transfer system, one of the main functions of the PLGA microspheres was to serve as the motion-controllable transporters in the RUF by which the encapsulated drug molecules were enabled to be brought to meet cells in particular sites in space (i.e., pressure nodes). Therefore, the CAPMs with d < 1 μm should be eliminated because they cannot be immobilized at pressure nodes since their movement will be primarily driven by the microstreaming drag force instead of the primary acoustic radiation force in the RUF [25]. Combined with the fact that the efficiency of phagocytosis is highly dependent on the dimension of the polymeric microspheres used [26], the size of the CAPMs played a crucial role in this RUF-mediated drug delivery approach. In this study, the CAPMs with mean size of 2.23 ± 0.04 μm (Fig 3) enabled to provide the maximal efficacy of phagocytosis according to a previously published study [26].

Based on the temporal progression of cells-CAPMs combination under RUF exposure (Fig 5, A2 –H2), we demonstrated that both DH82 macrophages and CAPMs can be quickly immobilized/co-localized at pressure nodes within 20 sec of RUF irradiation. Although the nontoxicity of RUFs and/or acoustic radiation forces to mammalian cells has been identified in numerous studies [27, 28], information about how RUFs affect cells in the presence of polymeric microspheres is scarce. Likewise, CAPMs may be shed due to mechanical stress generated from the ultrasonic field (if there is any) and/or collided/compressed with other particles or cells during RUF exposure, leading to structural damage and loss of encapsulated model drug consequently. Therefore, the influence of RUF on cells and CAPMs should be evaluated before application to drug delivery. In this study, none of detrimental effect of RUF on the cellular viability (Fig 6a), growth rate (Fig 6b), and CAPM integrity (Figs 7 and 8) was found, indicating that the developed synthetic drug delivery system is harmless to both macrophages and PLGA microspheres. The possible interpretation for the nontoxicity of RUF is that according to the (Eq 1), the primary acoustic radiation force at each acoustic node is theoretically zero and therefore the impact of mechanical stress generated by the RUF on the co-localized cells and microspheres was minimal.

Since the aggregation of the microparticles under RUF irradiation progressed over time (Fig 5), it is foreseeable that the duration of RUF application plays an essential role in RUF-mediated CAPM delivery. Indeed, our data showed that the degree of cells-CAPMs aggregation was augmented along with increase of RUF exposure time throughout the time course (Fig 9), in which the efficiency of CAPM delivery was significantly enhanced by about 3 fold (*P* < 0.05) as the RUF exposure time was increased from 0 to 15 min (Fig 9c). We reasoned that this enhancement was resulted from the rapid co-localization of cells and CAPMs in space and that the longer the RUF irradiation was applied, the greater the contact opportunity between cells and CAPMs was able to be. This hypothesis was further verified by comparing the CAPM delivery rates of DH 82 macrophages treated by RUF with and without CAPMs (Fig 10). Our data clearly showed that the RUF cannot promote the capacity of particle internalization for macrophages, indicating that the RUF-led co-localization, rather than sonoporation, was the mechanism for the enhanced CAPM delivery efficiency.

In addition to the primary acoustic radiation force-led particle co-localization, the secondary acoustic radiation force also played an essential role in CAPM delivery particularly when the developed synthetic molecular transfer system is scaled up for use in practice. Knowingly a detrimental bulk-type streaming (i.e., Eckart streaming) will be arisen in large-scaled RUF [29] by which the efficacy of particle aggregation will be severely disturbed and resulted in diminished efficacy of drug transfer. In this study, particle agglomerates were consolidated by the secondary acoustic radiation force and therefore enabled to sustain the shock of interferences such as heat convection and/or microstreaming induced by RUF. Although the results obtained from the bench-scaled RUF offer a foundation for systemic scale-up, the effects of Eckart streaming on delivery of drug carriers in large-scale settings require further examinations and efforts are currently in progress.

In summary, we have developed a synthetic molecular transfer system for *in vitro* macrophage drug delivery upon applications of drug-loaded microspheres (i.e., CAPMs) and RUF operation. We reasoned that the success of the developed system was achieved by increasing the contact frequencies between cells and therapeutics as illustrated in Fig 1. Although strategies of co-localization for drug/gene delivery, such as immobilization of drug molecules on the cell culture substrate [30, 31] or use of various setups to increase the contact between cells and drug molecules [32, 33], have been widely reported in the past decades, to our knowledge, the methodology developed in this study was the first trial that we exploited RUF to precisely co-localize the target cells and drug microcarriers in 3D to enhance the efficiency of macrophage drug delivery. Since the aforementioned effectiveness was mainly contributed by the increased contact opportunities between cells and drug carriers, this methodology may be extensively utilized to deliver different types of drug carriers and/or payloads such as nucleic acids or proteins to macrophages. Overall, the developed molecular transfer system offers an effective, efficient, and scalable alternative for macrophage drug delivery.
